# Supplementary material for: Feedback between Population and Evolutionary Dynamics Determines the Fate of Social Microbial Populations
Source: PLoS Biol. 2013 Apr 30;11(4):e1001547. doi: 10.1371/journal.pbio.1001547 (PMC3640081; doi:10.1371/journal.pbio.1001547)
Supplement: Text S1 — Detailed description of the model and the parameters used in the simulation. (DOCX) [file pbio.1001547.s006.docx]

**SUPPORTING INFORMATION**

**Text S1**

**Mathematical Model:** Even in the absence of cheaters, the growth of cooperator cells is density dependent, and affected both by the Allee effect at low densities, and by logistic growth at higher densities. Previously [1,2], we have shown that a bi-phasic logistic model is able to not only fit experimental growth curves, but also to correctly predict the bifurcations of the system. The model is given by the following equation:

(1)

where *Nc* denotes cooperator density, *K* denotes the carrying capacity and *Ncrit* is the critical cooperator density where cooperators switch from the slow growth phase (characterized by a maximum per-capita growth rate of *γ_l_*) to the fast growth phase (characterized by a maximum per-capita growth rate of *γ_h_*).

Given the previously demonstrated success [1] of this very simple model to capture cooperator dynamics under very similar experimental conditions to the ones used in this work, we have extended the model to account for the presence of cheaters. When we have attempted to grow pure cheater cultures in our growth media, the cultures did not show any appreciable growth over a period of time of 23.5hrs. Therefore, we make the assumption that at low cooperator densities (*Nc<Ncrit*)*,* cheaters have a low growth rate per capita of *r_l_=γ_l_* (*1-a*), where *1a0*. Therefore, at low cooperator densities (*Nc<Ncrit*)*,* the coupled growth of cooperators and cheaters (whose density is represented by the variable *Nd*) is governed by the Lotka-Volterra equations, which represents the simplest density dependent model of inter and intra-specific competition [3]:

(2)

At high cooperator densities (*Nc>Ncrit*), cheaters do not have to pay the metabolic burden of making invertase, but they have access to the glucose made by the cooperators. This interaction can be modeled by assuming that the growth rate of cheaters (*r_h_*) is larger than that of cooperators (*γ_h_*) by a factor (*1+b*), where *b>0*, so that *r_h_=*(*1+b*) *γ_h_*. Finally, our model has to capture the fact that, at high densities, cooperators and cheaters also compete for all nutrients in the environment and are also described by a Lotka-Volterra model of competition [3]:

(3)

Finally, our model considers a lag phase of growth at the beginning, as the cells adapt to the new environment after they are diluted. This is modeled by assuming that no growth at all occurs for a time interval of duration *T_lag_* at the beginning of each growth cycle. The model was solved numerically using mathematica. The solutions were evaluated at *T=23.5hr*, thus obtaining the cooperator (*Nc*(*T*)) and cheater (*Nd* (*T*)) densities at the end of the growth cycle. The dilution process was simulated by dividing *Nc*(*T*) and *Nd* (*T*) by a fixed “dilution factor” (typically of 667x unless otherwise noted). The resulting densities of cooperators and cheaters after dilution were the starting point for a new growth cycle. The process was iterated multiple times, simulating a serial growth-dilution experiment. The parameters used in the simulations are given below, and represent estimations for the values that those parameters may take under the conditions of our experiment, and have been chosen so that they are consistent with previously measured growth rates and carrying capacities of yeast in sucrose [1,2,4]:

| **Parameter** | **Value in simulation** | **Reference** |
| --- | --- | --- |
| *γ_l_* | 0.31 hr^-1^ | Dai et al ^16^ |
| *γ_h_* | 0.47 hr^-1^ | Dai et al ^16^ |
| *Ncrit* | 276 μL^-1^ | Dai et al ^16^ |
| *T_lag_* | 3 hr | Dai et al ^16^ |
| *b* | 0.06 | This study |
| *a* | 0.075 | This study |
| *K* | 83,341 μL^-1^ | This study |

**References**

1. Dai L, Vorselen D, Korolev KS, Gore J (2012) Generic indicators for loss of resilience before a tipping point leading to population collapse. Science 336: 1175–1177. doi:10.1126/science.1219805

2. Celiker H, Gore J (2012) Competition between species can stabilize public-goods cooperation within a species. Mol. Syst. Biol. 8: 621. doi:10.1038/msb.2012.54

3. Hastings A (1997) Population Biology. Springer US.

4. Gore J, Youk H, Van Oudenaarden A (2009) Snowdrift game dynamics and facultative cheating in yeast. Nature 459: 253–256. doi:10.1038/nature07921
